# Untargeted serum metabolites profiling in high-fat diet mice supplemented with enhanced palm tocotrienol-rich fraction using UHPLC-MS

**DOI:** 10.1038/s41598-021-00454-9

**Published:** 2021-10-25

**Authors:** Danial Efendy Goon, Sharaniza Ab-Rahim, Amir Hakimi Mohd Sakri, Musalmah Mazlan, Jen Kit Tan, Mardiana Abdul Aziz, Norizal Mohd Noor, Effendi Ibrahim, Siti Hamimah Sheikh Abdul Kadir

**Affiliations:** 1 Institute of Medical Molecular Biotechnology (IMMB), Faculty of Medicine, Universiti Teknologi MARA (UiTM), Cawangan Selangor, Sungai Buloh, Selangor Malaysia; 2 Institute of Pathology, Laboratory and Forensic Medicine (I-PPerForM), Faculty of Medicine, Universiti Teknologi MARA (UiTM), Cawangan Selangor, Sungai Buloh, Selangor Malaysia; 3 Department of Biochemistry, Faculty of Medicine, Universiti Teknologi MARA (UiTM), Cawangan Selangor, Sungai Buloh, Selangor Malaysia; 4 Department of Physiology, Faculty of Medicine, Universiti Teknologi MARA (UiTM), Cawangan Selangor, Sungai Buloh, Selangor Malaysia; 5 Department of Biochemistry, Faculty of Medicine, Universiti Kebangsaan Malaysia, 56000 Kuala Lumpur, Malaysia; 6 Department of Pathology, Faculty of Medicine, Universiti Teknologi MARA (UiTM), Cawangan Selangor, 47000 Sungai Buloh, Selangor Malaysia

**Keywords:** Biochemistry, Diseases, Molecular medicine

## Abstract

Excessive high fat dietary intake promotes risk of developing non-alcoholic fatty liver disease (NAFLD) and predisposed with oxidative stress. Palm based tocotrienol-rich fraction (TRF) has been reported able to ameliorate oxidative stress but exhibited poor bioavailability. Thus, we investigated whether an enhanced formulation of TRF in combination with palm kernel oil (medium-chain triglycerides) (ETRF) could ameliorate the effect of high-fat diet (HFD) on leptin-deficient male mice. All the animals were divided into HFD only (HFD group), HFD supplemented with ETRF (ETRF group) and HFD supplemented with TRF (TRF group) and HFD supplemented with PKO (PKO group). After 6 weeks, sera were collected for untargeted metabolite profiling using UHPLC-Orbitrap MS. Univariate analysis unveiled alternation in metabolites for bile acids, amino acids, fatty acids, sphingolipids, and alkaloids. Bile acids, lysine, arachidonic acid, and sphingolipids were downregulated while xanthine and hypoxanthine were upregulated in TRF and ETRF group. The regulation of these metabolites suggests that ETRF may promote better fatty acid oxidation, reduce oxidative stress and pro-inflammatory metabolites and acts as anti-inflammatory in fatty liver compared to TRF. Metabolites regulated by ETRF also provide insight of its role in fatty liver. However, further investigation is warranted to identify the mechanisms involved.

## Introduction

Daily excessive high fat diet consumption increases serum free fatty acids (FFA) and cholesterol which is known as primary factor leading to high lipid deposition in the liver and eventually cause hepatic steatosis^1^. Non-alcoholic fatty liver disease (NAFLD) is a spectrum of liver disease covering from benign hepatic steatosis to many severe conditions such as non-alcoholic steatohepatitis (NASH), fibrosis and cirrhosis^2^. Based on the “multiple-hit hypothesis”, NAFLD begins with steatotic liver and may remain benign for a period but the condition promotes oxidative stress due to accumulation of intrahepatic lipid storage^3^. At the same time, increased serum FFA and cholesterol promote insulin resistance in NAFLD patients, leading to pro-inflammatory conditions in the liver and increased de novo lipogenesis (DNL), further increasing lipid storage^4,5^. The cascade of events repeats as a vicious cycle leading to higher lipid deposition in the liver resulting in severe hepatic steatosis.

Among the concerning aspect of NAFLD is the availability of effective pharmacotherapy agents in preventing the progression. The common pharmacotherapy options are anti-hyperglycemic agents such as metformin^6^, glucagon-like peptide-1 (GLP-1) receptor agonist^7^, sodium-glucose co-transporter 2 (SGLT2) inhibitors^8^ and thiazolidinediones^9^. Lipid lowering agents such as statins^10^ and nuclear receptors drugs such as ursodeoxycholic acid (UCDA)^11^ and obeticholic acid (OCA)^12^ are other choices included as a treatment for NAFLD. Despite the range of treatments available for this disease, these options exerted undesirable adverse effects. For example, metformin and GLP-1 induce diarrhoea^13,14^ while thiazolidinediones induce weight gain^15^. Other agents such as statins, UCDA and OCA have proven ineffective in treating NAFLD and far more suitable for NASH^10,16,17^.

Vitamin E has two isoforms which are tocopherol and tocotrienol. Tocotrienol supplementation for a year has been demonstrated to improve NAFLD by normalising liver echogenic findings and remissions were reported^18^. In another study, supplementation with tocotrienol for 12 weeks in NAFLD patients reported significant improvement of liver profile and inflammatory markers but liver echogenic findings remained unchanged^19^. On the other hand, TRF supplementation has been demonstrated to effectively prevent the progression of NAFLD in animal models^20–23^. Although metabolomic studies on TRF are limited, studies using Vitamin E consisting major portion of tocopherol demonstrated its increase in lysophosphatidylcholine of human plasma^24^. However, inconsistencies in NAFLD outcomes in human and animal studies in terms of antioxidant capabilities remained persistent.

Poor bioavailability of TRF may contribute to the inconsistencies among studies as explained by lower plasma tocotrienol concentration detected in humans compared to tocopherol^25,26^. It was reported that the half-life of tocotrienol was up to nine times shorter compared to tocopherol^27^. In addition, the bioavailability of each tocotrienol homolog differed when administered orally in rats and the bioavailability was much poorer when administered intravenously or intramuscularly^28^. Oral administration of TRF is provided in the form of a formulation that combines with a lipid carrier. The type of lipid-carrier for TRF plays a vital role in ensuring high bioavailability. Long-chain triglycerides (LCT) is commonly used as the carrier for TRF compared to medium-chain triglycerides (MCT). Apart from the length of fatty acids, the metabolic route taken by these triglycerides are non-similar. Once ingested, LCT is digested enzymatically before being absorbed by enterocytes and transported into the circulatory system. However, MCT has shown to be readily absorbed and transported directly into the liver through the portal vein to be further metabolised^29–31^.

Therefore, the supplementation of TRF in combination with MCT should be investigated using animal model. Apart from that, metabolomic analysis should be carried out to examine and provide knowledge relating to the role of TRF in modulating and regulating metabolites in NAFLD. Hence, in this study, an enhanced formulation of TRF (ETRF) comprising of higher percentage of tocotrienol (80%) combining with palm kernel oil (PKO), an MCT as the carrier was supplemented in mice provided with high-fat diet and the sera were subjected to untargeted metabolomics analysis using UHPLC-MS. This group was compared to TRF combined with palm oil as LCT carrier and another group subjected to high-fat diet (HFD) only as control.

## Results

### Differential metabolomic analysis between groups against HFD respectively

**Figure 1:**
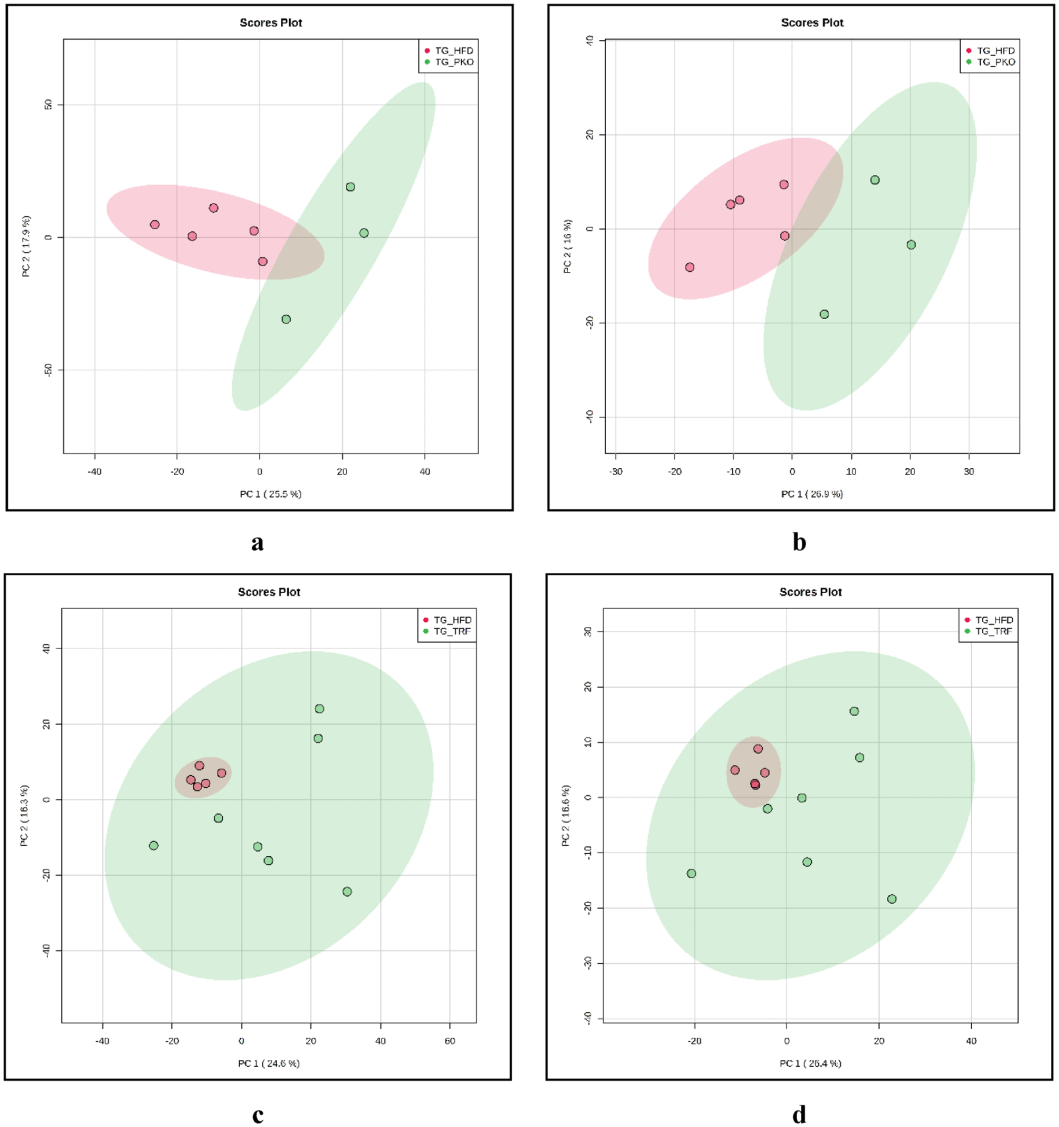

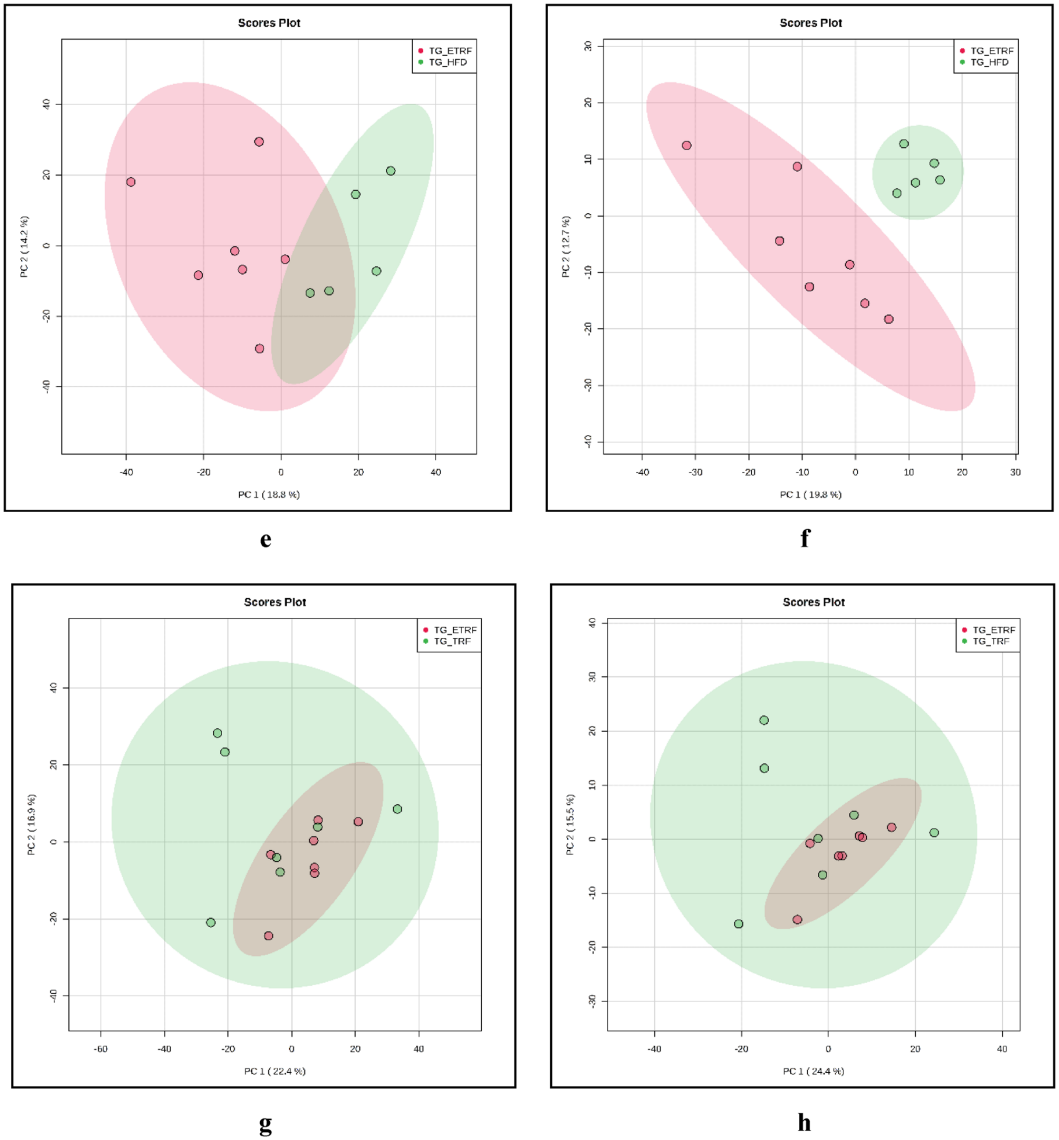
PCA score plots between PKO (green filled circle) and HFD (red filled circle) in positive mode (**a**) and negative mode (**b**), PCA score plot between TRF (green filled circle) and HFD (red filled circle) in positive mode (**c**) and in negative mode (**d**), PCA score plot between ETRF (red filled circle) and HFD (green filled circle) in positive mode (**e**) and negative mode (**f**), PCA score plot between ETRF (red filled circle) and TRF (green filled circle) in positive mode (**g**) and negative mode (**h**).

Principal Component Analysis (PCA) of PKO against HFD generated a total of 20,832 peaks in positive mode with average of 2604 peaks per sample. The generated peaks per groups were 2060. The PCA score plot generated showed 43.4% variation of PKO when compared to HFD in the positive mode whereby the first principal component (PC1) score was 25.5% and second principal component (PC2) score was 17.9% (Fig. 1a). The PCA of PKO against HFD in negative mode generated a total peak of 10,136 peaks with 1267 peaks average peaks per sample and total peak groups generated were 1001. The PCA score plot for negative mode described variation of 42.9% with 26.9% of PC1 and 16% of PC2 (Fig. 1b). Univariate analysis of PKO against HFD showed 101 metabolic features were detected in positive mode (Supplementary Fig. 1a) and 38 in negative mode (Supplementary Fig. 1b). The total of metabolic features from both positive and negative mode was 139.

Principal Component Analysis (PCA) of TRF against HFD generated a total peak of 31,248 peaks in positive mode with average of 2604 total peaks per sample and 2060 peak groups formed. The PCA score showed total variation of 40.9% with PC1 at 24.6% and PC2 at 16.3% on positive mode when compared to HFD (Fig. 1c). The PCA in negative mode of TRF against HFD generated a total peak 15,204 in which the average peaks per sample were at 1267 peaks and total peak groups formed were 1001. The PCA score of generated a total variation of 43% where PC1 score was 26.4% and PC2 score was 16.6% (Fig. 1d). Univariate analysis of TRF group against HFD detected 141 metabolic features in positive mode (Supplementary Fig. 1c) and 55 metabolic features in negative mode (Supplementary Fig. 1d). This gave rise to the total of 196 metabolic features.

The total peaks generated from PCA of ETRF against HFD in positive mode were 31,248 peaks where each sample achieved average of 2604 peaks and peak groups formed were 2060. The PCA showed total variation of 33% (Fig. 1e) where PC1 score was 18.8% and PC2 score was 14.2%. The PCA in negative mode of ETRF against HFD generated a total peak of 15,204 peaks where average peaks per sample were 1267 and peak groups formed were 1001. The PCA plot showed complete separation in PCA with total variation of 32.5% (Fig. 1f) where PC1 described 19.8% variation and 12.7% for PC2. Univariate analysis of ETRF against HFD resulted in total of 239 metabolic features identified using volcano plot. This include 156 metabolic features in positive mode and 83 metabolic features were identified in negative mode. The plot is shown in Supplementary Fig. 1e and Supplementary Fig. 1f, respectively.

In addition, PCA generated a total of 36,456 peaks in positive mode when ETRF was compared against TRF where the average peaks per sample were 2604 and the total peak groups were 2060. The PCA score showed total of 39.3% variation in positive mode (Fig. 1g) where PC1 score was 22.4% and PC2 at 16.9%. The PCA of ETRF against TRF in negative mode generated 17,738 total peaks which average peaks per sample were 1267 and the total peak groups generated were 1001. The PCA score in negative mode showed total variation of 39.9% with PC1 score of 24.4% and PC2 of 15.5% (Fig. 1h). Univariate analysis of ETRF group against TRF showed 42 metabolic features were detected in positive mode and 23 metabolic features in negative mode. Total metabolic features identified were 65. The volcano plot for positive mode and negative mode are illustrated in Supplementary Fig. 1g and Supplementary Fig. 1h, respectively.

### Metabolites profiling and regulation in treatment against control group

Treatment group consisting of ETRF, TRF and PKO groups were compared against HFD group, the control group respectively. In PKO group, a total of 33 metabolites were successfully identified from 139 metabolic features (Table 1). These metabolites belong to group of alkaloids, fatty acids, bile acids, lipids, and amines. Xanthine was found to be the most up-regulated and deoxyadenosine monophosphate was the most down-regulated metabolites.

**Table 1:** List of metabolites in PKO against HFD.

Metabolite	Fold-change (log^2^)^a^	*p* value
Xanthine	8.4072	0.032212
Isovaltrate	7.5962	0.000106
Limonin	5.3958	0.001841
(15Z)-9,12,13-Trihydroxy-15-octadecenoic acid	1.7977	0.0164
Sphinganine	1.2211	0.00767
Xanthosine	1.1539	0.040012
Spermidine	1.0461	0.049502
N-Methylhistamine	0.924	0.027424
13,14-Dihydro PGF2a	0.91792	0.0459
Taurochenodeoxycholic acid	0.8734	0.029087
D-Sphingosine	0.81567	0.043147
Nicotinamide	0.80644	0.047193
L-Glutamic acid	0.79069	0.038329
indospicine	0.77237	0.007492
Dehydrofelodipine	− 0.60594	0.034791
3-Ethoxyandrosta-3,5-dien-17beta-ol propanoate	− 0.82539	0.047198
Dihydrolipoate	− 0.94479	0.003521
6-Methoxyquinoline	− 0.98664	0.013298
D-Glucurono-6,2-lactone	− 1.0511	0.034869
Nicotine glucuronide	− 1.0877	0.008214
3-Indoxyl sulphate	− 1.0989	0.009656
Netilmicin	− 1.2984	0.038169
Sulfoglycolithocholic acid	− 1.5375	0.020523
Cholic acid	− 1.6234	0.025529
Cyclic-3,20-bis(1,2-ethanediyl acetal)-11alpha-(acetyloxy)-5alpha,6alpha-epoxypregnane-3,20-dione	− 1.7427	0.006786
Oleamide	− 1.9727	0.041883
Hexanoylcarnitine	− 2.2494	0.01046
C16 Sphinganine	− 2.2925	0.002023
2-amino-hexadecanoic acid	− 3.1642	0.000117
Phytosphingosine	− 4.2454	0.000344
gamma-Glutamylcysteine	− 5.4559	2.20 × 10^−7^
N-Undecanoylglycine	− 6.0083	2.25 × 10^6^
Deoxyadenosine monophosphate	− 7.0388	3.23 × 10^−6^

^a^Fold change values described are the ratio of PKO/HFD. The values are arranged from highest ratio (up-regulated) to the lowest (down-regulated).

In TRF supplemented group, 42 metabolites were identified from a total of 196 metabolic features (Table 2). Here, the profiled metabolites consisted of lipids, alkaloids, and bile acids. Isovaltrate was found to be the most up-regulated while N-Undecanoylglycine was the most down-regulated when compared against HFD group.

**Table 2:** List of metabolites in TRF against HFD.

Metabolite	Fold-change (log^2^)^a^	*p* value
Isovaltrate	7.6429	0.00149
( +)-abscisic acid beta-D-glucopyranosyl ester	5.9806	7.22E−05
Paucin	5.1866	4.45E−06
2,2-dichloro-1,1-ethanediol	4.0956	0.045523
Mupirocin	3.8462	0.030777
Taurochenodeoxycholic acid	3.8371	0.019658
L-Olivosyl-oleandolide	3.7225	0.030254
Taurohyocholic acid	3.6062	0.019682
Taurocholic acid	3.2801	0.023508
Ouabain	2.9975	0.02805
pseudaminic acid	1.8028	0.006537
(15Z)-9,12,13-Trihydroxy-15-octadecenoic acid	1.5964	0.005335
indospicine	1.286	0.000424
N-Acetyl-ala-ala-ala-methylester	1.0483	0.009132
3-propylmalic acid	0.86307	0.008511
Palmitoyl sphingomyelin	0.62613	0.041855
Trigonelline	0.59162	0.029791
Glycerophosphocholine	− 0.59431	0.029666
11-amino-undecanoic acid	− 0.60037	0.0468
Z-Arg-Arg-NHMec; Benzyloxycarbonylarginyl-arginine 4-methylcoumarin-7-ylamide	− 0.62836	0.035491
Artocarpin	− 0.68462	0.008561
lysophosphatidylethanolamine 0:0/20:4(8Z,11Z,14Z,17Z)	− 0.70745	1.69E−05
Biotin sulfone	− 0.73369	0.029679
C16 Sphingosine-1-phosphate	− 0.74896	0.021534
Glycerophospho-N-Oleoyl Ethanolamine	− 0.81377	0.000621
1-octadecylglycero-3-phosphocholine	− 0.82645	0.046111
4-Pyridoxic acid	− 0.86159	0.007487
Arachidonic acid	− 0.87795	0.030662
2-Hydroxy-4-methylthiobutanoic acid	− 0.88465	0.020117
DL-Lysine	− 0.88836	0.000675
Allantoic acid	− 0.89861	0.000472
Istamycin C1	− 1.1599	0.010525
D-Sphingosine	− 1.3232	0.001413
O-6-deoxy-a-L-galactopyranosyl-(1- > 3)-O-b-D-galactopyranosyl-(1- > 3)-O-2-(acetylamino)-2-deoxy-b-D-glucopyranosyl-[1- > 3(or 1- > 6)]-O-[O-b-D-galactopyranosyl-(1- > 4)-2-(acetylamino)-2-deoxy-b-D-glucopyranosyl-[1- > 6(or 1- > 3)]]-O-b-D-galactopyranosyl-(1- > 4)-D-G	− 1.3292	0.016392
Oleamide	− 1.3343	0.038229
Netilmicin	− 1.3959	0.001809
3-Phenyllactic acid	− 1.4653	0.011921
N-(5Z,8Z,11Z,14Z-docosatetraenoyl)-ethanolamine	− 1.4942	0.003432
Hexadecanamide	− 1.6835	0.025216
C16 Sphinganine	− 2.4489	9.82E−06
2-amino-hexadecanoic acid	− 2.7841	2.10 × 10^−6^
Phytosphingosine	− 4.9224	6.48 × 10^−8^
N-Undecanoylglycine	− 6.1852	1.11 × 10^−9^

^a^Fold change values described are the ratio of TRF/HFD. The values are arranged from highest ratio (up-regulated) to the lowest (down-regulated).

Out of the 239 metabolic features identified in the ETRF supplemented group, 40 metabolites were successfully identified (Table 3). Similar as to profiled metabolites in PKO group, metabolites profiled for ETRF comprised of alkaloids, fatty acids, bile acids, lipids, amines, and amino acids. Xanthine was found to be the most up-regulated in this group followed by (+)-abscisic acid beta-D-glucopyranosyl ester. The most down-regulated profiled metabolite was phytosphingosine.

**Table 3:** List of metabolites in ETRF against HFD.

Metabolite	Fold-change (log^2^)^a^	*p* value
Xanthine	9.2854	0.014729
( +)-abscisic acid beta-D-glucopyranosyl ester	7.239	4.26E−14
Paucin	5.5155	8.23E−10
Hypoxanthine	4.2737	0.023667
Urocanic acid	1.0146	0.003096
L-Ascorbic acid 2-sulfate	0.81939	0.042157
3-Isopropylmalic acid	0.66469	0.029228
6-Methylnicotinamide	0.6507	0.023537
Trigonelline	0.64591	0.003216
PE(22:6(4Z,7Z,10Z,13Z,16Z,19Z)/0:0)	− 0.59609	0.00128
Indolelactic acid	− 0.61605	0.003202
Ethylenediaminetetraacetic acid (EDTA)	− 0.61975	0.014081
LysoPE(0:0/18:2(9Z,12Z))	− 0.62167	0.017277
PC(20:4(5Z,8Z,11Z,14Z)/0:0)	− 0.65119	0.014296
L-Threonine	− 0.66945	0.003803
6′-[(carboxymethyl)-C-hydroxycarbonimidoyl]-2′,3′,4,4′,5,6-hexahydroxy-[1,1′-biphenyl]-2-carboxylic acid	− 0.68279	0.016133
C16 Sphinganine	− 0.70745	1.69E−05
LysoPE(0:0/20:4(5Z,8Z,11Z,14Z))	− 0.70745	1.69E−05
Butenylcarnitine	− 0.71444	0.034775
3-Indoxyl sulphate	− 0.73989	0.029273
Pyridoxamine	− 0.76347	0.003376
Arachidonic acid	− 0.76829	0.002173
DL-ß-Leucine	− 0.7949	0.01281
C16 Sphingosine-1-phosphate	− 0.80979	0.002821
(9S,10S)-10-hydroxy-9-(phosphonooxy)octadecanoic acid	− 0.8574	0.004992
L-Acetylcarnitine	− 0.86237	0.011089
LysoPE(18:1(9Z)/0:0)	− 0.88265	5.92E−06
DL-Lysine	− 0.88836	0.000675
Allantoic acid	− 0.89861	0.000472
4-Pyridoxic acid	− 0.98191	0.001064
Sphinganine	− 1.0196	0.015898
3-Ethoxyandrosta-3,5-dien-17beta-ol propanoate	− 1.0514	0.049831
N-(5Z,8Z,11Z,14Z-docosatetraenoyl)-ethanolamine	− 1.0554	0.007735
Netilmicin	− 1.1707	0.01109
Cyclic-3,20-bis(1,2-ethanediyl acetal)-11alpha-(acetyloxy)-5alpha,6alpha-epoxypregnane-3,20-dione	− 1.317	0.025216
D-Sphingosine	− 1.3232	0.001413
Cholic acid	− 1.486	0.029476
3-Phenyllactic acid	− 1.5077	0.0071
2-amino-hexadecanoic acid	− 2.7841	2.10 × 10^−6^
Phytosphingosine	− 4.9224	6.48 × 10^−8^

^a^Fold change values described are the ratio of ETRF/HFD. The values are arranged from highest ratio (up-regulated) to the lowest (down-regulated).

Apart from comparison between treatment groups with control group, ETRF group was also compared against TRF group. The total metabolites identified when ETRF was compared against TRF were 15 from the total of 65 metabolic features (Table 4). From 15 of successfully profiled metabolites, four belong to bile acids metabolism. Other metabolites profiled fall under fatty acids and amino acids. Similar to comparison of ETRF against HFD, the comparison of the former against TRF also showed that (+)-abscisic acid beta-D-glucopyranosyl ester was the most upregulated. The most downregulated metabolite profiled was 2,2-dichloro-1,1-ethanediol.

**Table 4:** List of metabolites in ETRF against TRF.

Metabolite	Fold-change (log^2^)^a^	*p* value
( +)-abscisic acid beta-D-glucopyranosyl ester	1.2584	0.010707
Succinyladenosine	0.74051	0.038448
(3beta,24R,24'R)-fucosterol epoxide	0.70934	0.00632
N6,N6,N6-Trimethyl-L-lysine	0.66537	0.040981
N-Acetyl-D-quinovosamine; 2-Acetamido-2,6-dideoxy-D-glucose	0.59846	0.035022
Tuliposide B	− 0.89808	0.027011
N-Acetyl-1-aspartylglutamic acid	− 1.2084	0.012566
Ouabain	− 3.0274	0.009455
Taurocholic acid	− 3.2595	0.008659
Mupirocin	− 3.3253	0.029892
Taurochenodeoxycholic acid	− 3.4266	0.014632
taurohyocholic acid	− 3.5021	0.008178
Sulfoglycolithocholic acid	− 3.5678	0.013053
L-Olivosyl-oleandolide	− 3.761	0.009798
2,2-dichloro-1,1-ethanediol	− 4.4637	0.010266

^a^Fold change values described are the ratio of ETRF/TRF. The values are arranged from highest ratio (up-regulated) to the lowest (down-regulated).

Profiled metabolites that were similar across groups were identified and were compared based on fold-change. The comparison was made between PKO (Table 1), TRF (Table 2) and ETRF (Table 3) groups against HFD, respectively. Based on these comparisons, the major metabolite group involved is bile acid. Cholic acid was found to be downregulated in ETRF and PKO group. Taurochenodeoxycholic acid was found to be upregulated in TRF and PKO. However, bile acids such as Taurocholic acid, Taurochenodeoxycholic acid, Taurohyocholic acid and Sulfoglycolithocholic acid were all downregulated in ETRF when compared to TRF.

Another metabolite group detected across the groups are amino acids. DL-Lysine was found downregulated in ETRF and TRF when compared to HFD group. The amino acid 2-amino-hexadecanoic acid was also found to be downregulated in ETRF, TRF and PKO. Apart from that, the amino acid N-Undecanoylglycine was downregulated in TRF and PKO. However, indospicine was found to be upregulated in TRF and PKO. When comparing to TRF, the amino acid N6, N6, N6-Trimethyl-L-lysine was found to be upregulated in ETRF. The derivative of aspartic acid, Succinyladenosine was found to be upregulated in ETRF as well.

Fatty acids were also found to be regulated by the groups. Arachidonic acid was downregulated in ETRF and TRF group. Oleamide was also found to be downregulated in TRF and PKO. The fatty acid (15Z)-9,12,13-Trihydroxy-15-octadecenoic acid was found to upregulated in TRF and PKO. Isovaltrate was found to be upregulated in TRF and PKO as well. However, comparison between ETRF and TRF, Tuliposide B, a fatty acid derivative was found to be downregulated in the former.

Lipids and sphingolipids were another major metabolite group found to be regulated. The lipid N-(5Z,8Z,11Z,14Z-docosatetraenoyl)-ethanolamine was found to be downregulated in ETRF and TRF. Another lipid detected such as Cyclic-3,20-bis (1,2-ethanediyl acetal)-11alpha-(acetyloxy)-5alpha,6alpha-epoxypregnane-3,20-dione was downregulated in both ETRF and PKO. C16 Sphinganine was found to be downregulated in ETRF, TRF and PKO. Similarly, Phytosphingosine was also found downregulated in ETRF, TRF and PKO. C16 Sphingosine-1-phosphate was downregulated in ETRF and TRF.

Other significant different metabolites across the groups were identified. Organic acid such as 3-Phenyllactic acid was found to be downregulated in both ETRF and TRF. Alkaloids were also found to be regulated. Xanthine was found upregulated in ETRF and PKO. Hypoxanthine was found to be upregulated in ETRF only. The alkaloid trigonelline was found upregulated in ETRF and TRF.

### Biochemical pathways analysis of metabolites profiled in ETRF against TRF

**Figure 2:**
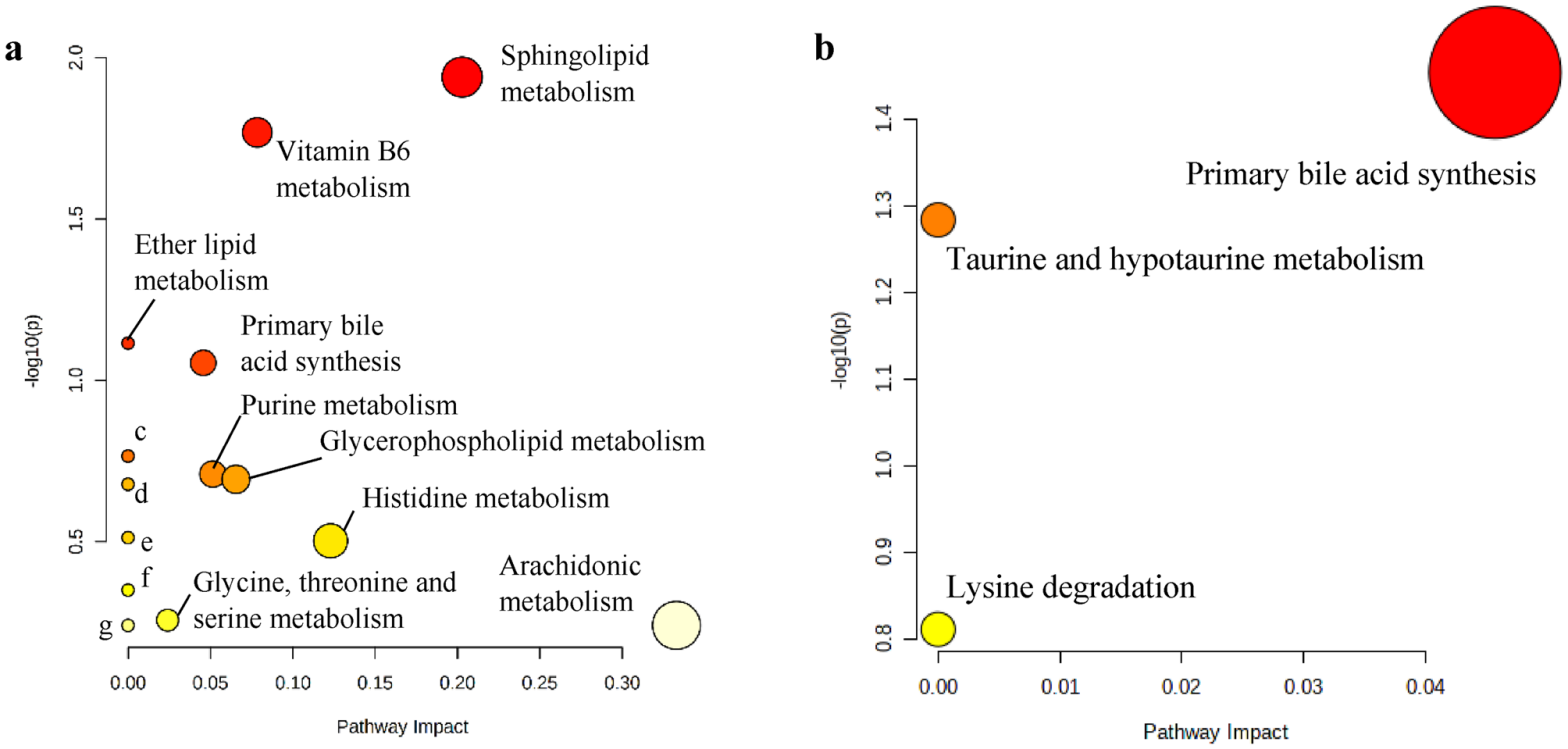
Biochemical pathways identified in TRF and ETRF against HFD (**a**) and ETRF against TRF (**b**) using MetaboAnalyst. Analysis of the metabolites profiled in TRF and ETRF against HFD generated 15 biochemical pathways while the metabolites profiled in ETRF against TRF derived three biochemical pathways that were altered. c: taurine and hypotaurine metabolism; d: biotin metabolism; e: aminoacyl-tRNA metabolism; f: lysine degradation; g: biosynthesis of unsaturated fatty acids.

Pathway analysis using MetaboAnalyst on the metabolites profiled in TRF and ETRF against HFD generated total of 15 biochemical pathways (Table 5). Significant biochemical pathways detected were Sphingolipid and Vitamin B6 metabolism (Fig. 2a) (*p* < 0.05). Analysis of the metabolites profiled in ETRF against TRF showed three biochemical pathways that were altered. The pathways involved were primary bile acid synthesis, taurine and hypotaurine metabolism and lysine degradation (Fig. 2b). From these pathways, primary bile acid synthesis was found to be significant (*p* < 0.05) (Table 6).

**Table 5:** List of biochemical pathways (MetaboAnalyst) identified in TRF and ETRF against HFD.

Pathway name	*p*-value*	− log(p)	Impact
Sphingolipid metabolism	0.011484	1.9399	0.20284
Vitamin B6 metabolism	0.017056	1.7681	0.07843
Ether lipid metabolism	0.07676	1.1149	0.0
Primary bile acid biosynthesis	0.088346	1.0538	0.0457
Valine, leucine and isoleucine biosynthesis	0.17185	0.76485	0.0
Taurine and hypotaurine metabolism	0.17185	0.76485	0.0
Purine metabolism	0.1954	0.70907	0.05145
Glycerophospholipid metabolism	0.20287	0.69279	0.0655
Biotin metabolism	0.21011	0.67755	0.0
Aminoacyl-tRNA biosynthesis	0.30767	0.51191	0.0
Histidine metabolism	0.31487	0.50187	0.12295
Lysine degradation	0.44715	0.34955	0.0
Glycine, serine and threonine metabolism	0.55447	0.25612	0.02408
Biosynthesis of unsaturated fatty acids	0.57542	0.24002	0.0
Arachidonic acid metabolism	0.57542	0.24002	0.33292

The asterisk denoted significant value of p less than 0.05 (*p* < 0.05) for statistical analysis.

**Table 6:** List of biochemical pathways (MetaboAnalyst) identified in ETRF against TRF.

Pathway name	*p*-value*	− log(p)	Impact
Primary bile acid biosynthesis	0.035146	1.4541	0.0457
Taurine and hypotaurine metabolism	0.052021	1.2838	0.0
Lysine degradation	0.15456	0.81089	0.0

*p-value of less than 0.05 is regard as significant (*p* < 0.05).

### The effect of TRF and ETRF on liver histology and Farnesoid-X receptor (fxr) expression

The effect of TRF and ETRF were assessed on each liver histologically and stratified using NAFLD Activity Score (NAS)^32^. The scoring was assessed based on the presence of macrovesicular and microvesicular steatosis, lobular inflammation and fibrosis. Scoring for each group is outlined in Table 7. Results from NAS showed that more than half of TRF group (57%) had mid-grade steatosis with 52% of livers sampled presented with Zone 3 steatosis. On the other hand, ETRF presented with about 90% had high grade steatosis and 81% with Zone 3 steatosis. Fibrosis was not found in TRF while only 5% presented with perisinusoidal fibrosis. In terms of lobular inflammation, about 67% in TRF group presented with < 2 foci of lobular inflammation and 24% with > 2 foci of lobular inflammation. In contrast, 57% from ETRF presented with < 2 foci of lobular inflammation while 38% presented with no inflammation.

**Table 7:** Assessment of NAFLD activity score (NAS).

Item	Definition	Group			
		HFD (%)	PKO (%)	TRF (%)	ETRF (%)
Steatosis	< 5%	0	0	0	0
	5–33%	0	0	5	0
	> 33–66%	5	24	57	10
	> 66%	95	76	38	90
Location	Zone 3	29	33	52	81
	Zone 1	0	10	0	0
	Azonal	14	10	19	5
	Panacinar	57	57	29	14
Fibrosis	None	91	86	100	95
	Perisinusoidal or periportal	9	14	0	5
	Perisinusoidal and portal/periportal	0	0	0	0
	Bridging fibrosis	0	0	0	0
	Cirrhosis	0	0	0	0
Lobular inflammation	No foci	71	67	9	38
	< 2 foci	29	33	68	57
	2–4 foci	0	0	24	5
	> 4 foci	0	0	0	0
Hepatocyte ballooning	None	100	100	100	100
	Few balloon cells	0	0	0	0
	Prominent ballooning	0	0	0	0

**Figure 3:**
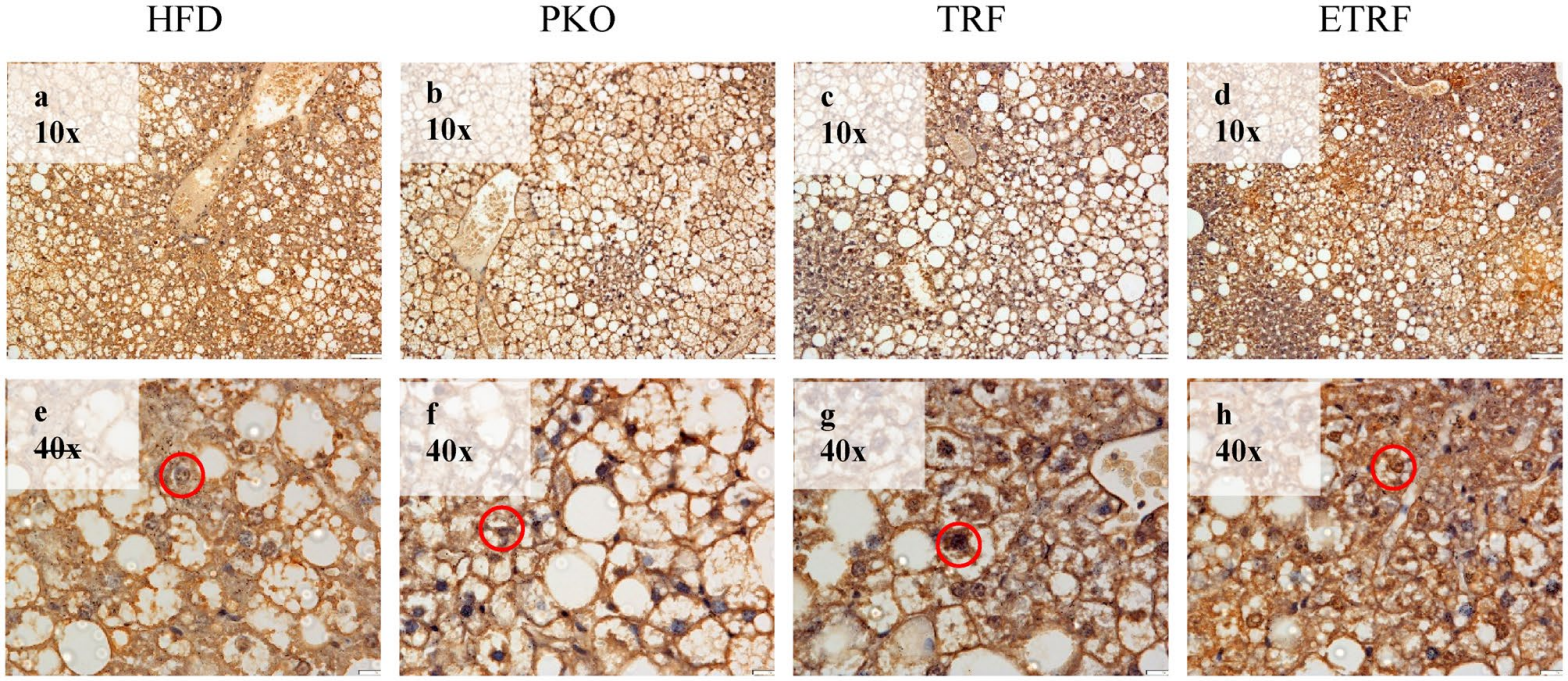
The effect of HFD, PKO, TRF and ETRF on Farnesoid-X Receptor (fxr) expression based on immunohistochemistry (IHC). Representative histology images of IHC staining of liver fxr in HFD (**a**,**f**), PKO (**b**,**g**), TRF (**c**,**g**) and ETRF (**d**,**h**) at 10 × (**a**–**d**) and 40 × (**e**–**h**) magnifications. Expression of fxr is demarcated by a red circle where expression was assessed based on positive cells identified by the intensity of intracellular chromogen (brown) staining and number of hepatocytes expressing the staining which could be observed at 40 × magnification.

**Figure 4:**
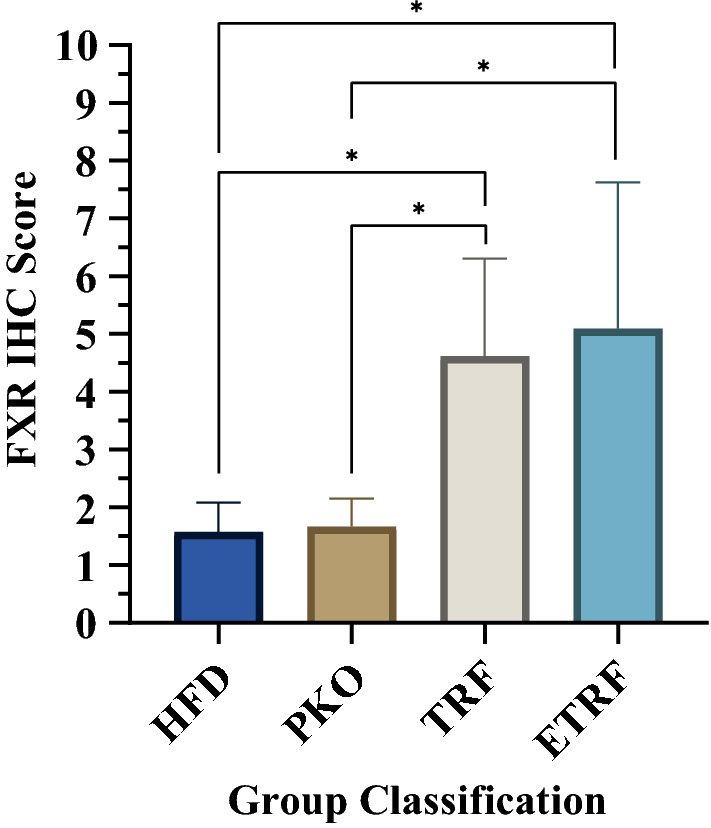
Farnesoid-X Receptor (fxr) immunohistochemistry (IHC) scoring based on IHC Scoring. Expression of fxr is significant with TRF when compared to HFD and PKO, respectively. Expression of fxr was also significant with ETRF when compared to HFD and PKO, respectively (*p < 0.05). Comparison between TRF and ETRF was not significant (*p* > 0.05). Data is expressed as mean of scoring ± standard deviation (SD). (n = 7).

Liver sampled from each mouse from HFD, PKO, TRF and ETRF were assessed on their fxr expression using immunohistochemistry (IHC) technique. The expression was measured using IHC scoring method^33–35^. The representative of IHC staining from each group is shown in Fig. 3 where the expression of fxr is demarcated by red circle. The scoring of fxr expression is shown in Fig. 4. The highest fxr score was found in ETRF (5.1 ± 0.55) followed by TRF (4.62 ± 0.37), PKO (1.67 ± 0.11) and HFD (1.57 ± 0.11). Statistical analysis showed TRF and ETRF were significantly higher compared to PKO and HFD respectively (*p* < 0.05).

### The effect of TRF and ETRF on liver Farnesoid-X receptor (*fxr*) and its target genes, small heterodimer partner (*shp*) and signal transducer and activator of transcription 3 (*stat3*)

**Figure 5:**
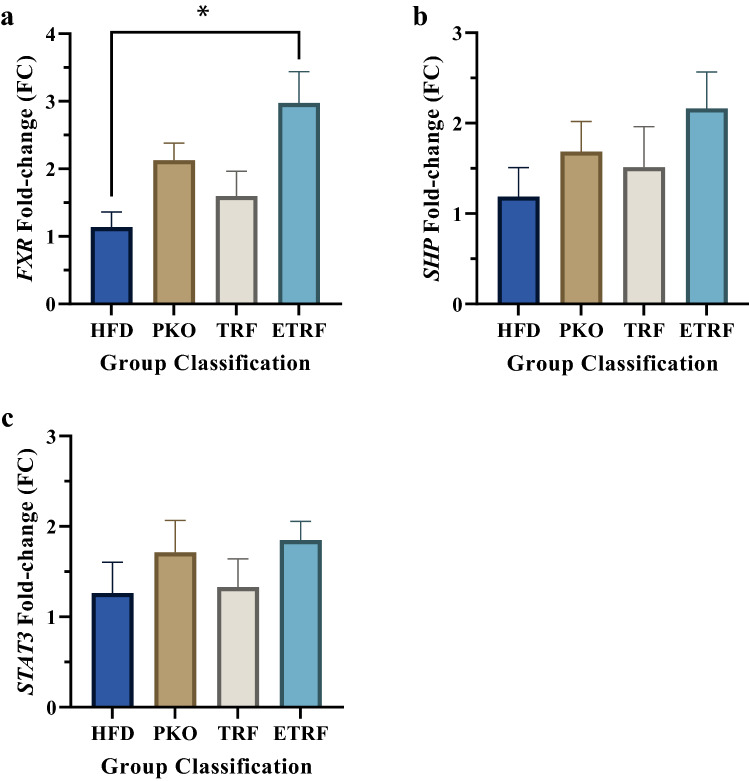
The effect of PKO, TRF and ETRF on liver *fxr* (**a**), *shp* (**b**) and *stat3* (**c**) expressions based on fold-change (FC). The FC of liver *fxr* was found significant in ETRF compared to HFD but not with *shp* and *stat3*. The expression of *fxr* and its target genes, *shp* and *stat3* were found to be not significant in PKO and TRF. (n = 7) (*p < 0.05).

The fold-change (FC) of liver Farnesoid-X Receptor (*fxr*) gene expression was highest in ETRF (2.98 ± 0.46) followed by PKO (2.13 ± 0.25), TRF (1.60 ± 0.36) and HFD (1.14 ± 0.64). The expression of *fxr* was found to be significant in ETRF when compared to HFD (*p* < 0.05) (Fig. 5a). Gene expression of small heterodimer partner (*shp*) was found highest in ETRF (2.16 ± 0.40) followed by PKO (1.69 ± 0.33), TRF (1.51 ± 0.45) and HFD (1.19 ± 0.32). There was no significant difference between groups in *shp* gene expression (*p* > 0.05) (Fig. 5b). Similarly, signal transducer and activator of transcription 3 (*stat3*) gene expression was found highest in ETRF (1.85 ± 0.21) followed by PKO (1.71 ± 0.35), TRF (1.33 ± 0.35) and HFD (1.26 ± 0.34) (Fig. 5c). Statistical analysis showed no difference between groups (*p* > 0.05).

## Discussion

This study reported untargeted metabolomic analysis of enhanced formulation of TRF (ETRF) supplementation in mice fed with HFD (60% kcal from fat) using the genetically modified B6.Cg-LepOb/J strain. This mice model was used as it has been reported as an appropriate model to study fatty liver since the leptin-deficiency was able to withheld from involvement of inflammation without the “second-hit”^36^. Gogiashvili et al.^37^ has also reported that the development of fatty liver in similar strain being fed with HFD (60% kcal from fat). In this study, ETRF group was compared to HFD group and TRF group respectively to provide insight of the different metabolites being regulated by each supplementation. The ETRF carrier, PKO was also compared to HFD group in terms of the difference in metabolite regulations. The source of TRF utilised in this study was derived from Malaysian palm tree *Elaeis guineensis*. The palm-based TRF from this tree has been investigated of their therapeutic properties primarily in Malaysia as it is being readily accessible while Western countries have inclination towards annatto oil or rice bran oil-based TRF. The difference of these oils are the percentages of Vitamin E homologs contained in these oils. Annatto and rice bran oil-based TRF contain highest concentration of δ-tocotrienol and minimal amount of tocopherol^23,38,39^. On the other hand, palm oil, both its seed and kernel possessed tocotrienol and tocopherol in which each homolog is relatively of the same concentration^40^. Therefore, investigation utilises palm-based TRF may provide robust information in metabolomic analysis as the comprehensive content of tocotrienol and tocopherol would affect multiple genes and metabolic pathways^41^.

Our study has detected both primary and secondary bile acids (BAs), in conjugated or unconjugated form, in all treatment groups when compared to HFD group. In ETRF group, cholic acid (CA), a primary BA, was found to be downregulated. In contrast, TRF group showed upregulation of primary BA, taurochenodeoxycholic acid (TCDA). Meanwhile, the PKO groups demonstrated both profiles seen in TRF and ETRF, whereby CA was downregulated and TCDA was upregulated. In addition, comparing ETRF to TRF, both primary and secondary BAs which are taurocholic acid (TCA), TCDA, taurohyocholic acid (THCA) and sulfoglycolithocholic acid were downregulated in the former. In NAFLD, it was reported that the synthesis and reuptake of BAs were disrupted^42,43^. Studies have reported that both primary and secondary BAs synthesis were increased but hepatic bile signaling was suppressed^44,45^. Apart from that, study by Tang et al.^46^ and Kodama et al.^47^ reported that rats with hepatic steatosis showed upregulation of TCA and downregulation of hyodeoxycholic acid and taurohyodeoxycholic acid. Our findings suggested that the ETRF supplementation, as opposed to TRF, most likely to regulate and improve bile acids synthesis in fatty liver. The downregulation of these primary and secondary BAs detected in ETRF was perhaps explained by the ability of its gamma tocotrienol in suppressing the expression of cytochrome P450 7A1 (CYP7A1), a key enzyme for synthesis of primary bile acids^48^. Apart from that, Farnesoid-X Receptor (fxr), a key receptor in bile acid regulation, was reported repressed in NAFLD^49^. In our result, liver fxr protein and gene expressions were shown to be highest in ETRF suggesting that CA downregulation may occur as result of a negative feedback mechanism. The negative feedback mechanism occurred by the increase in fxr expression leading to increase in *shp* gene expression. The increase in *shp* promotes repression of BAs reuptake transporter such as Na^+^ taurocholate cotransporting polypeptide (NTCP) and suppression of CYP7A1^50^. On the other hand, TRF findings were similar but the fold changes are lower than ETRF. This could be the reason why TRF unable to improve BAs regulations which resulted in spilling of the BAs into the circulation as profiled in the metabolomic data. The outcome observed with ETRF supplementation was most likely contributed by the use of MCT carrier. MCT would promote direct absorption into the liver by bypassing the vascular circulation, allowing high bioavailability of ETRF promoting higher activation of fxr and repressing BAs synthesis while LCT require catabolism and packaging, in process, lower fxr activation and BAs synthesis repression.

The metabolites profiled in ETRF and TRF have detected metabolites that are responsible in promoting inflammation or act as anti-inflammatory. Pro-inflammatory metabolites such as arachidonic acid and sphingolipids were found to be downregulated in both ETRF and TRF. Anti-inflammatory metabolite such as trigonelline was found to be upregulated in both ETRF and TRF when compared against HFD.

Arachidonic acid was found downregulated in both ETRF and TRF group. Arachidonic acid is metabolised by the cyclooxygenase pathway and lipoxygenase pathway. Study by Hall et al.^51^ demonstrated that remodeling of phospholipid membrane occurred in NAFLD whereby release of arachidonic acid from the membrane promote cell injury and inflammation. In another study, individuals with NAFLD were reported to express high level of 5- hydroxyeicosatetraenoic acid (5-HETE) and 9- hydroxyoctadecadienoic acid (9-HODE), product of arachidonic acid metabolism^52^. It was also reported that NAFLD has higher level of leukotrienes and lower level of eicosanoid^53^. HFD animals supplemented with ETRF or TRF exhibited reduction of arachidonic acid would most likely result from the supplement’s antioxidant activities and preventing remodeling of phospholipid membrane in hepatocytes. Apart from that, it was also reported that Vitamin E has capability in modulating arachidonic acid oxidation by inhibiting 5-lipoxygenase activity^54^. Inhibition of the enzyme would result in inhibition of leukotrienes synthesis, preventing inflammatory response in the liver. In addition, the gene expression of Signal Transducer and Activator of Transcription 3 (*stat3*) was detected to increase in ETRF and TRF despite not being significant. Although increase in *stat3* has shown to accelerate fibrosis^55^ and γ-tocotrienol has been demonstrated to prevent fibrosis by suppressing *stat3* by inhibiting its phosphorylation^56^. However, paradoxical effect from independent increase of *stat3* was observed to promotes anti-inflammatory properties in NAFLD^57,58^. Therefore, it is most likely that the increase in *stat3* in NAFLD as observed in this study promoted by ETRF and TRF. Further investigation should be performed in order to elucidate the paradoxical mechanism.

In our study, sphingolipids such as sphinganine and phytosphinganine were found to be downregulated in all groups when compared to HFD group. Sphingolipid signaling mediator, sphingosine-1-phosphate (S1P), was found to be downregulated in ETRF and TRF. Sphingolipids were reported to play role in pathogenesis of NAFLD. Gorden, et al.^59^ reported that sphingolipids such as ceramides, dihydroceramides and 1-deoxy- dihydroceramides were increased in NAFLD and NASH. Studies have reported that caloric restriction and vitamin E supplementation were able to reduce sphingolipids level and prevent progression of NAFLD by suppression of mRNA expression of ceramide^60,61^. Similar to our findings, downregulation of sphingolipids, primarily sphinganine and phytosphinganine most likely occurred in the same mechanism. S1P is a signaling molecule and its formation occurs by enzymatic reaction of sphingosine kinase on sphingosine^62^ and has role in progression of NASH^63^. S1P activates sphingosine-1-phosphate receptor (S1PR) especially S1PR2 which highly expressed in the liver, responsible in mass cell degranulation and inflammatory response^64,65^. Hence, ETRF and TRF showed downregulation of S1P which may promote lower inflammation in the liver. Apart from that, S1P signaling was reported to be closely related to bile acids signaling. It was reported that bile acid, along with S1P, activates S1PR2 to regulate hepatic glucose and lipid metabolism through ERK1/2 and AKT signaling pathways^66–68^. Therefore, although ETRF and TRF exhibited similar capacity to normalise sphingolipids in NAFLD, ETRF most likely to have robust properties by further decreasing the level of S1P, which would prevent the progression of NAFLD to NASH.

In this study, the antioxidant trigonelline was found to be upregulated in both ETRF and TRF when compared to HFD. The plant alkaloid trigonelline is a nicotamide that is commonly found in coffee and plants^69^. Trigonelline supplementation was reported to promote protective effect against NAFLD^70^. Similarly, study has reported that supplementation of trigonelline in NAFLD model was able to normalise liver function readings and increase the expression of Bcl-2 protein and decrease Bax protein expression^71^. However, in this study, the source of trigonelline would be most likely derived within tocotrienol-rich fraction but not within the carrier. This is because the alkaloid was undetected in PKO. However, further investigation is warranted to identify the presence of this alkaloid in palm oil. The upregulation of trigonelline in both ETRF and TRF group when compared to HFD suggest that the presence of this alkaloid may prevent steatosis in these group. Histologic assessment of the liver would be able to support this finding. Comparison based on fold-change between ETRF and TRF showed that the alkaloid is upregulated higher in the former group providing insight that ETRF may have higher.

Our study found that N-undecanoylglycine, a N-fatty acylglycine was downregulated in TRF and PKO. However, the metabolite was not observed in ETRF. N-Undecanoylglycine, an acylglycine is a derivative of glycine and undecanoic acid have shown to be involved in fatty acid oxidation^72^. Study by Liu et al.^73^ and Du et al.^74^ have demonstrated that N-Undecanoylglycine was upregulated in hepatocytes subjected to oxidative stress or alteration in lipid metabolism occurred. Fatty acids that are unable to be further oxidised will lead to accumulation due to mitochondrial damage in NAFLD. The accumulation would involve microsomal ω-oxidation commonly occur in animal or peroxisome β-oxidation in human by conjugating fatty acid with glycine^75^. Fatty acid oxidation that takes place in peroxisome would increase the expression of L-carnitine in order to increase shuttling of fatty acid into peroxisome to overcome the burden of excessive fatty acid deposition^76^. However, the fatty acid oxidation is not as effective as mitochondrial β-oxidation. In TRF and PKO, the downregulation of N-Undecanoylglycine suggested that the fatty acid oxidation in these groups have been improved compared to HFD. This was perhaps due to inhibition of de novo lipogenesis (DNL) protein expression^77^ activation of PPARα receptor and gene expression of Carnitine Palmitoyltransferase 1A (CPT1A) and CYP3A4^38,78,79^. On the other hand, ETRF showed undetected N-Undecanoylglycine which may suggest that fatty acid oxidation in this group has improved due to its MCT carrier to increase its bioavailability. This is further supported by the downregulation of L-acetylcarnitine and upregulation of N6, N6, N6-Trimethyl-L-lysine. L-acetylcarnitine has shown to inversely reflect the capacity of mitochondrial β-oxidation^80,81^. Apart from that, the increase in carnitine synthesis was reported to be increased with N6, N6, N6-Trimethyl-L-lysine^82^. Based on the regulations of these metabolites, it is suggested that β-oxidation has been increased in ETRF compared to TRF and HFD group.

Amino acids that are involved in fatty acid oxidation showed alteration in regulation of metabolites in ETRF and TRF group when compared to HFD. The amino acid lysine was detected downregulated in both ETRF and TRF group. These amino acids are known as ketogenic amino acids that form acetoacetate and acetyl-CoA^83^. Previous studies have reported that lysine was found high in serum of NAFLD patients^84,85^ and in animal model^86^. It was also reported that increase in urine lysine and leucine concentration are associated with NASH^87,88^ which similarly observed in NAFLD animal model^89,90^. These ketogenic amino acids were demonstrated to be regulated by silent mating type information regulation 2 homolog 5 (SIRT5)^91^ where previous study has shown that SIRT5 was downregulated in NAFLD leading to increase in serum lysine level^92^. Apart from that, ketogenesis is shown to be favored as source of energy in NAFLD due to suppression of β-oxidation^93^. Therefore, the downregulation of these ketogenic amino acids in both ETRF and TRF suggested that the source of energy in these groups favored towards improved β-oxidation. However, compared to TRF, additional downregulation of lysine detected in ETRF suggested that better β-oxidation occurred in this group and reduce the reliance of these amino acids as source of energy. However, further investigation is required to further support these findings in order to elucidate the roles of ETRF and TRF on ketogenic pathway in NAFLD.

Xanthine was detected to be upregulated in ETRF and PKO. The precursor of xanthine, hypoxanthine was identified to be upregulated in ETRF. Product of xanthine metabolism such as allantoic acid was detected to be downregulated in both ETRF and TRF group when compared to HFD. In human, the catabolism of hypoxanthine and xanthine ended with uric acid. However, in animal, the end product of xanthine metabolism is allantoic acid^94^. Study has reported that the level of serum uric acid was higher in NAFLD patients^95^. In animal model, similar findings have been reported^96,97^. The metabolites are known to be involved in purine salvage pathway. In our study, the precursor of uric acid, xanthine and hypoxanthine were upregulated in ETRF and PKO but uric acid was not detected in the serum metabolomics analysis. This positive outcome is perhaps contributed by ETRF and PKO respectively. However, ETRF group may had additional benefits from the combination with MCT. Studies have demonstrated that palm oil-based vitamin E was able to reduce plasma xanthine oxidase activities which may prevent hyperuricemia^98,99^. Studies have also reported that dietary MCT was able to prevent hyperuricemia by reducing xanthine oxidase activity but the underlying mechanism require further investigation^100,101^. Nevertheless, the presence of high amount of uric acid in serum inferred from the increased level of xanthine may not always be pathological. Uric acid may be regarded as antioxidant in serum, not in cytoplasm^102^.

Although both ETRF and TRF possessed all the Vitamin E homologs, the higher percentage of tocotrienol in ETRF and the use of MCT to increase its bioavailability will be able to provide the findings discovered in this study. However, further investigations are required to fully deduce these findings. Investigation at genomic and proteomic levels would provide the answer in confirming the expression of metabolites involved. Information regarding the effect of ETRF and TRF on the gene and protein expression could be inferred as well. Apart from that, targeted metabolomic analyses based on findings in this study would validate these findings. Based on the PCA score plot, the comparison between PKO and ETRF with HFD respectively provides distinct differences between the groups, especially in ETRF group negative mode, which presented with clear separation between groups. Therefore, lipidomic analysis in this model will provide valuable and important information on lipids metabolism that plays a significant role in fatty liver and information on lipids regulated by ETRF and TRF. Among the limitation faced in this study was from technical perspective. The B6.Cg-LepOb/J mice were obese and had cause a challenge in cardiac blood aspiration upon euthanising. This occurred due to the heart displacement from the excessive intraperitoneal fat deposition. Therefore, it is recommended to perform the aspiration by utilising ultrasound probe or handled by a highly trained personnel in blood collection.

This study has investigated the metabolites regulated in fatty liver induced by HFD in B6.Cg-LepOb/J mice subjected to ETRF supplementation. Metabolomic analysis showed supplementation of ETRF in mice with fatty liver suggested promotion of β-oxidation, reduction in oxidative stress and inflammation through downregulation of primary and secondary bile acids, lysine, arachidonic acid and sphingolipids. Supplementation of ETRF has shown its capabilities in mediating the activity of signaling molecule in several pathways. This includes bile acids synthesis, fatty acid β-oxidation, glycine, serine and lysine metabolism, sphingolipids metabolism and arachidonic acid metabolism. Apart from that, the supplementation also exhibited antioxidant activities though xanthine and trigonelline upregulation. TRF supplementation may exhibit similar capacity to ETRF when compared against HFD but lacking additional properties such as inability to exert as signaling molecule in carnitine pathway and sphingosine-1-phosphate signaling.

## Methods

### Tocotrienol-rich fraction

Tocotrienol-rich Fraction (TRF; Tri.E™), Enhanced TRF (ETRF; Gold Tri.E™), stripped palm oil (PO) and stripped palm kernel oil (PKO) were gifts from Sime Darby Oils (Selangor, Malaysia). The composition of Tri.E™ was 72% tocotrienol and 28% tocopherol. In Gold Tri.E™, the percentage of tocotrienol was 81% and the remaining was tocopherol. The TRF was prepared by mixing with PO (long-chain triglycerides; LCT) at the ratio of 1:1. ETRF was prepared by mixing with PKO (medium-chain triglycerides; MCT) with the same ratio.

### Animal study

This animal study was approved by the Universiti Teknologi MARA (UiTM) Animal Care Unit Committee (ACUC) [approval number: UiTM CARE 4/2018 (251/2018)] and conducted according to the Animal Welfare Act 2015 (Act 772), Law of Malaysia. This study is reported in accordance with the ARRIVE guidelines^103^. In this study, fatty liver was induced using genetically modified mice through high-fat diet (60% kcal from fat). Twenty-eight B6.Cg-LepOb/J male mice at 8 weeks old were purchased from Jackson Laboratory (Maine, USA). The sample size was calculated using G*Power^104^ calculator with the determined by the statistical test to be ANOVA with the parameters of effect size (E) of 0.9 and alpha (α) of 0.05, giving rise to the total sample of 28 (n = 28). The age of mice represented adult human age as reported by Dutta and Sengupta^105^. These mice were caged individually with unlimited supply of reverse-osmosis water in room with regulated temperature of 24 ± 2° C and 12-h light/dark cycle in the Laboratory Animal Care Unit (LACU), UiTM, Sungai Buloh Campus. Mice were randomly selected and distributed evenly into four groups. No inclusion or exclusion criteria were applied on mice during grouping. Each group consisted of seven mice (n = 7). The groupings were based on the type of treatment they received during the study. The first group was control group whereby they received high-fat diet (HFD; 60% kcal from fat; Altromin, Lage, Germany) ad libitum. The second group was the carrier group in which the mice were provided HFD ad libitum and PKO, the carrier for ETRF at dose of 200 mg/kg daily. The third group was TRF group where the mice were provided with HFD ad libitum and 200 mg/kg daily supplementation of TRF. Lastly, the fourth group comprised of ETRF group. The group was provided ad libitum supply of HFD and daily supplementation of ETRF at dose of 200 mg/kg daily. The study was conducted for 6-weeks. At the end of 6-weeks, the mice were euthanised by rapid cervical dislocation. This method was selected as the use of chemicals to euthanise would affect the outcome of serum metabolomics analysis. Blood was collected in each mouse by cardiac aspiration using 26 G needle and 1 mL syringe. Collected blood was transferred into red-top blood tube (Becton Dickinson, USA). No mice were excluded at the end of study. However, due to technical challenge, the total number of blood able to be collected from the mice were seven for ETRF (n = 7), seven for TRF (n = 7), three for PKO (n = 3) and five for HFD (n = 5). Blood tubes were allowed to sit in room temperature for 20 min. Minimal of three biological replicates are adequate in metabolomics study^106^. Sera were acquired by centrifuging at 1500 × *g*, 4° C for 10 min. The serum appeared as supernatant in each tube was transferred into 2 mL microtubes and was stored in − 80 °C until required for analysis. Liver from each mouse was collected for histology assessment by fixing in 10% neutral buffered formalin (NBF) (R&M Chemicals, Malaysia) prior to processing.

### Serum preparation for metabolite extraction

Collected sera stored in − 80 °C were thawed and 100 µL of serum was transferred from each tube into another individual 2 mL microtube. Then, 300 µL of methanol (Optima® LC/MS, Fisher Chemical, USA) was pipetted into the microtube containing 100 µL of serum. The serum and methanol were vortexed for 15 s followed by centrifuging at 4 °C at 15,800 × *g* for 15 min. The supernatant was pipetted out into a new 2 mL microtube. The supernatants collected from all samples were dried using concentrator for 4 h using VAQ mode (Concentrator Plus, Eppendorf, Germany).

### UHPLC-MS

Dried sera were reconstituted using 100 µL water (W6-4 Water, Optima® LC/MS, Fisher Chemical, USA). The mixture was vortexed for 15 s and filtered with a 0.22 µm regenerated cellulose membrane. The mixture was transferred into glass vial. Blank samples consisted of 200 µL water was also prepared into the glass vials. The samples and blanks were arranged into the UHPLC autosampler (UltiMate™ 3000, Thermo Scientific™, USA). A C18 column (100 mm × 2.1 mm, 1.7 µm; Synchronis™, Thermo Scientific™, USA) was placed in the UHPLC system. The mobile phase A comprised water with 0.1% formic acid and mobile phase B comprised acetonitrile (ACN) with 0.1% formic acid. The chromatographic separation was set at a flow rate of 450 µL per minute and column temperature was set at 55° C. Injection volume of 2 µL was selected. A set of elution gradients was set at 0.5% B for 1 min, 0.5–99.5% B for 15 min, 99.5% B for 4 min, and 99.5–0.5% B for 2 min and blank sample was injected 15 times, followed by QC and serum samples on the autosampler. Untargeted metabolomics analysis was carried out using UltiMate™ 3000 and Q-Exactive HF Orbitrap-MS (Thermo Fisher Scientific, USA). The instrument was set up to analyse at 50 arbitrary unit (AU) sheath gas flow rate, 18 AU auxiliary gas flow rate, 0 AU sweep gas flow rate, 55 AU S-lens, 320 °C capillary temperature, 300 °C auxiliary gas heater temperature, spray voltage of 3.5 kV for positive mode and 3.0 kV for negative mode. Resolution of 60,000 with scan range of 100–1000 (*m/z*) was set for MS scanning followed by MS/MS scans at resolution of 15,000 with stepped normalised collision energy of 20, 40, and 60 AU. Spectral data acquired from the scanning were pre-processed using Xcalibur™ version 3.1 (Thermo Fisher Scientific, USA).

### Statistical analysis

Statistical analysis was conducted by comparing PKO, TRF and ETRF group against HFD group respectively. ETRF group was also compared against TRF. The total number of samples analyzed for serum metabolomics were seven for ETRF (n = 7), seven for TRF (n = 7), three for PKO (n = 3) and five for HFD (n = 5). Data normalisation was performed using MetaboAnalyst^107^. Chemometric and univariate analyses were also performed using MetaboAnalyst^107^. Principal Component Analysis (PCA) was selected as the tool of chemometric analysis to visualise the difference and outliers of the groupings in the form of score plot. Univariate analysis using volcano-plot was performed following PCA scoring by using parameter of fold-change of 1.5 and *p*-value less than 0.05 (*p* < 0.05). List of metabolic features generated from volcano plot was further identified using m/zcloud and chemspider included in Xcalibur™ version 3.1 (Thermo Fisher Scientific, USA), CEU Mass Mediator (CEUMM)^108^, Human Metabolome Database (HMDB)^72^ and METLIN.

### Histology assessment

The fixed livers collected were sampled from three main lobes giving total of 21 samples per group. After fixation in NBF for 72-h, all liver tissues were processed by the tissue processor (Sakura, Europe). Then, tissues were embedded into histology cassette using a paraffin-embedding machine (Thermo Scientific, Germany). The embedded tissues were allowed to cool for 24 h at room temperature and sectioned at 5 µm thick. The tissues were stained with Hematoxylin and Eosin (H&E; Merck, Germany) and Masson Trichrome (MT; Bio-Optical, Italy). Immunohistochemistry staining using fxr antibody was performed using the formalin fixed paraffin embedded liver tissues prepared prior. Tissue section of 5 μm thick was fixed on polylysine histology glass slide (Thermo Scientific, USA). Antigen retrieval was performed using Antigen Retrieval Solution (R&D System, USA) and IHC was carried out using HRP-DAB tissue staining kit (R&D System, USA) and fxr antibody (R&D System, USA). All tissue sections were assessed by resident histopathologists via single blinded assessment. The assessment of fxr by histopathologist was carried out using IHC Scoring Method^33–35^ comprising of assessing the positive cell and intensity of staining.

### RNA extraction

RNA was extracted from mice liver tissue using GeneJET RNA Purification Kit (Thermo Scientific, USA) following the protocol provided. Liver at approximately 30 mg was homogenised and supernatant collected were subjected to RNA collection and purification. Purified RNA was analysed using Quickdrop spectrophotometer to check for concentration and purity.

### First strand cDNA synthesis

CDNA was synthesized from RNA using Maxima First Strand cDNA Synthesis Kit for RT-qPCR (Thermo Scientific, USA). Master mix was prepared accordingly for each sample and control comprised of blank sample were incorporated in each session to assess signs of contamination. The microcentrifuge tubes are then placed into a thermal cycler with the following protocol:10 min at 25 °C, 15 min at 50 °C, 5 min at 85 °C. The acquired cDNA was aliquot to final concentration of 10 ng/µL.

### RT-qPCR

Quantitative reverse transcription polymerase chain reaction was carried out on a total of 28 RNA samples using Maxima SYBR Green qPCR Master Mix (2X) (Thermo Scientific, USA). The list of primers used are listed in Table 8. Target gene comprised of *fxr*, *shp* and *stat3*. The house-keeping gene comprised of *gapdh*, *itih4* and *ambh*. Prepared samples were subjected to RT-qPCR using Bio-Rad CFX 96 and were analysed using Bio-Rad CFX Maestro software. The protocols used are listed in Table 9. The annealing temperature varies according to the optimal melting temperature for each primer. Data were tabulated into excel and the expression of target genes are being quantified relative to the housekeeping genes.

**Table 8:** List of primers sequences used for RT-qPCR.

	Primer	Forward sequence	Reverse sequence
Target gene	*fxr*	5′-CTC AAG TTC TTC AGC CAC AGA-3′	5′-AGA TGC CAG GAG AAT ACC-3′
	*stat3*	5′-CTT CTC CTT CTG GGT CTG	5′-GCT CCT TGC TGA TGA AAC-3′
	*shp*	5′-ATC TCT TCT TCC GCC CTA TC-3′	5′-GTC ACC TCA GCA AAA GCA-3′
Reference gene	*gapdh*	5′-TGC ACC ACC AAC TGC TTA G-3′	5′-GGA TGC AGG GAT GAT GTT C-3′
	*itih4*	5′-GCA TCT ATG AGG ATT CAG ACT-3′	5′-ATG AGA GCA GTG GAT TGG-3′
	*ambh*	5′-ACC CTC AAG AAA GAA GAC-3′	5′-GCC GTT GTA GTA CCT-3′

**Table 9:** RT-qPCR protocol.

Step	Temperature, °C	Time	Number of cycles
UDG pre-treatment	50	2 min	1
Initial denaturation	95	10 min	1
Denaturation	95	15 s	40
Annealing	60	60 s	
Melt Curve	65 to 95	0.05 s	1

### Statistical analysis of group comparison

Non-parametric analysis using Kruskall-Wallis was selected to compare means between groups for IHC score and gene fold-change. *P*-value of less than 0.05 (*p* < 0.05) was regarded as statistically significant.

## Supplementary Information

Supplementary Information 1.
