# Supplementary material for: Untargeted serum metabolites profiling in high-fat diet mice supplemented with enhanced palm tocotrienol-rich fraction using UHPLC-MS
Source: Sci Rep. 2021 Oct 25;11:21001. doi: 10.1038/s41598-021-00454-9 (PMC8546078; doi:10.1038/s41598-021-00454-9)
Supplement: Supplementary file 1 — Supplementary Information 1. [file 41598_2021_454_MOESM1_ESM.docx]

| 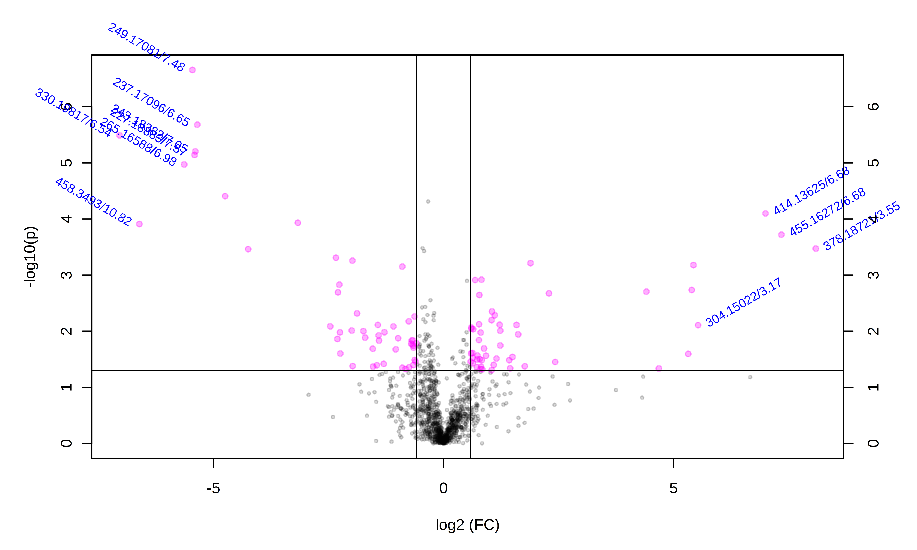 |
| --- |
| **a** |
| 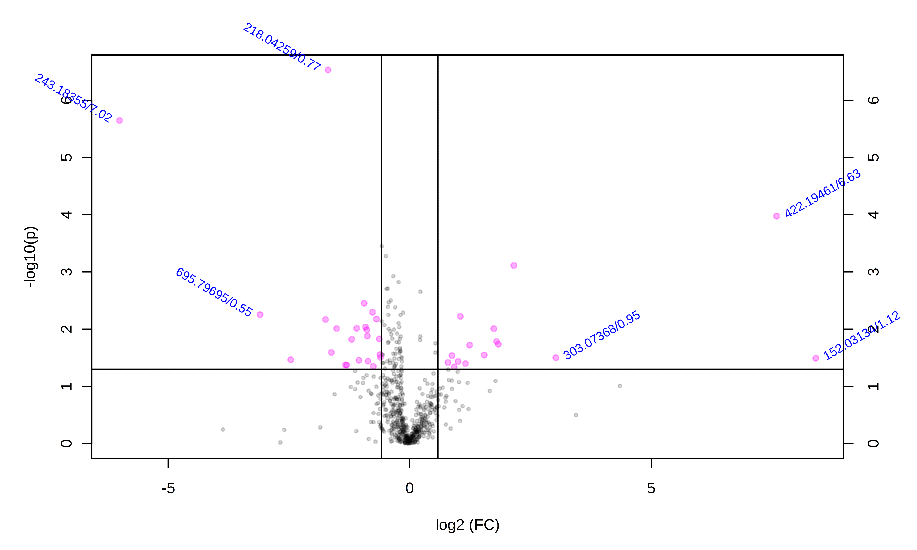 |
| **b** |

**Supplementary Figure 1**

| 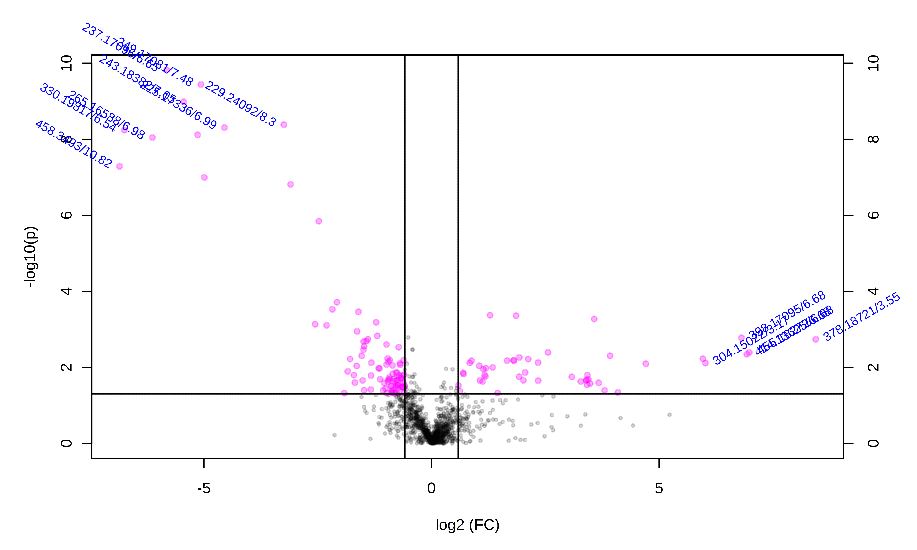 |
| --- |
| **c** |
| 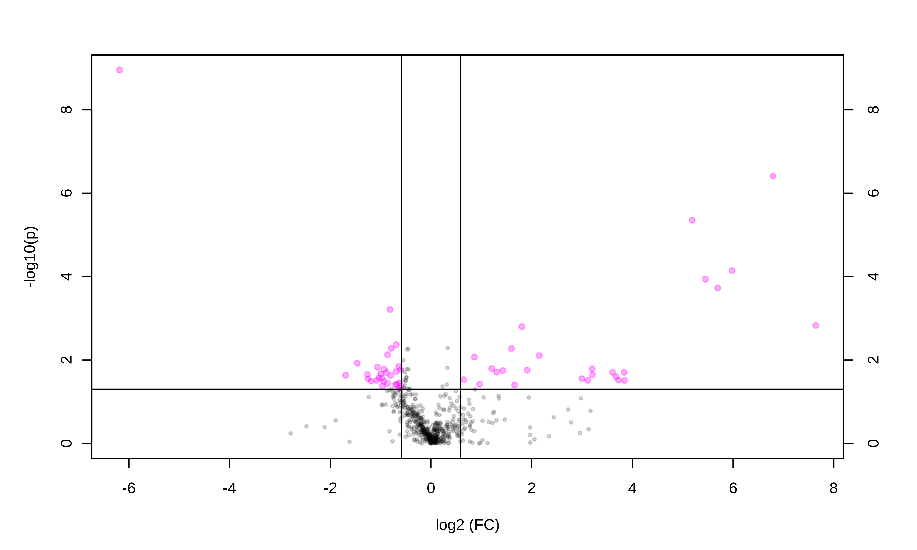 |
| **d** |
| 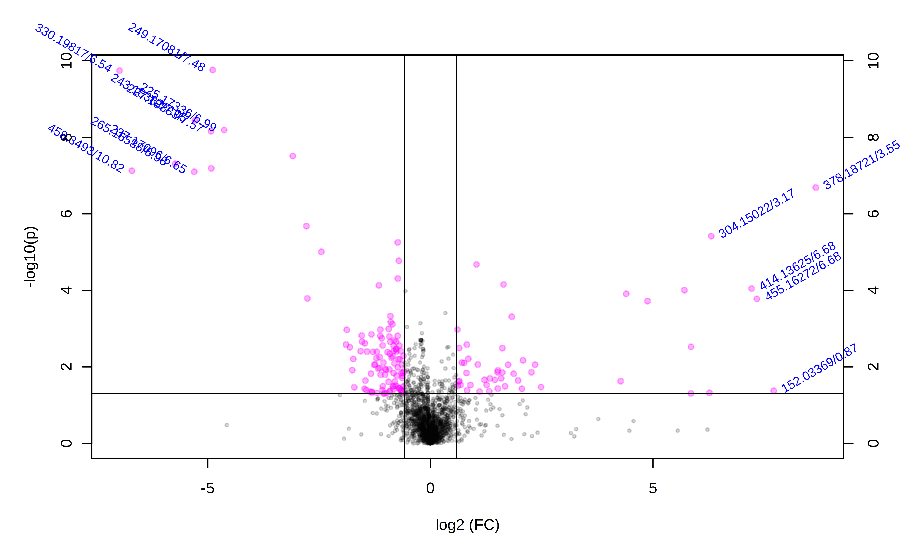 |
| **e** |
| 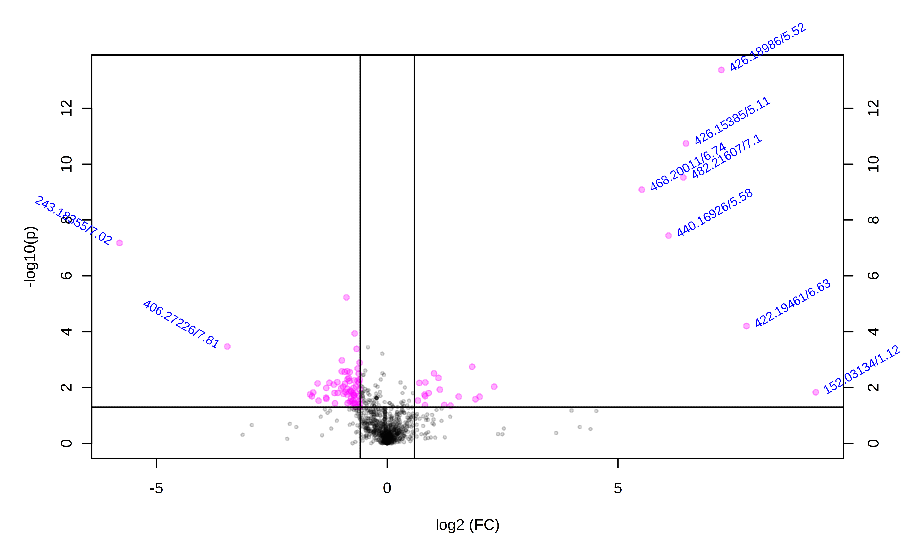 |
| **f** |

| 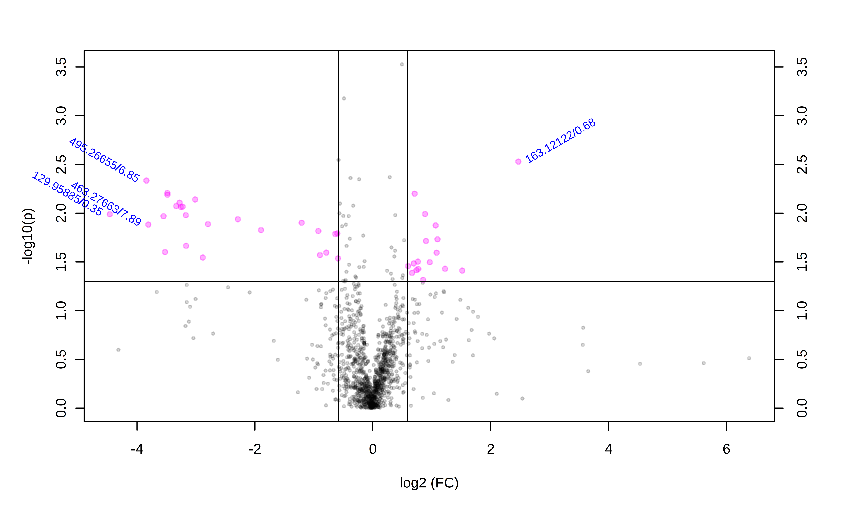 |
| --- |
| **g** |
| 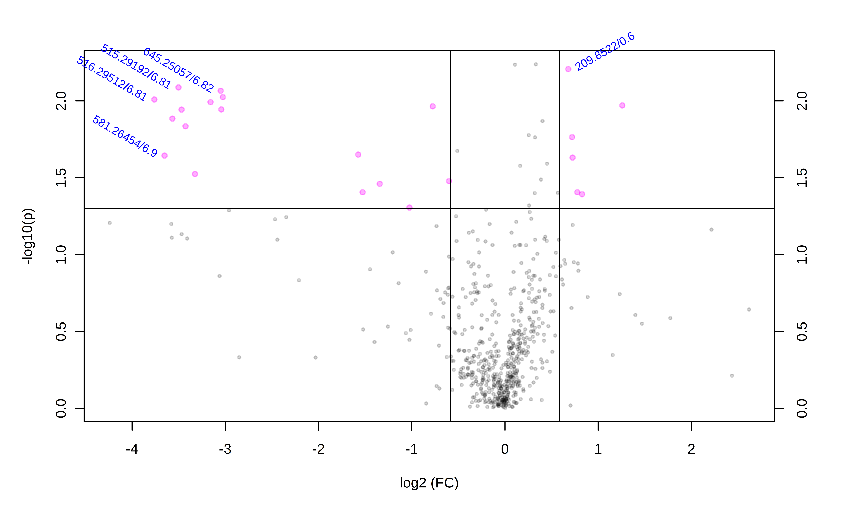 |
| **h**  **Supplementary Figure 1.** Volcano plot of metabolic features based on fold-change of 1.5 and p-value less than 0.05 of PKO against HFD where plot (a) represents the positive mode while plot (b) represents the negative mode. Plot (c) represents the positive mode while plot (d) represents the negative mode in TRF against HFD. Plot (e) represents the positive mode while plot (f) represents the negative mode in ETRF against HFD. Plot (g) represents the positive mode while plot (h) represents the negative mode in ETRF against TRF. |

Supplementary Data 1 – Biometric Data

Mean weight of B6.Cg-LepOb/J mice and standard deviation according to group and week of study.

| Week of study | Group | | | |
| --- | --- | --- | --- | --- |
|  | HFD (n=7) | PKO (n=7) | TRF (n=7) | ETRF (n=7) |
| 1 | 47.57 ± 2.070 | 49.00 ± 5.477 | 54.14 ± 3.761 | 47.86 ± 6.594 |
| 6 | 58.00 ± 1.915 | 57.57 ± 2.440 | 57.00 ± 3.000 | 57.14 ± 3.288 |

Note: mean body weight of mice is expressed as gram (g).

Measurement, mean and standard deviation of B6.Cg-LepOb/J mice waist circumference at the end of study

| Sample | Group | | | |
| --- | --- | --- | --- | --- |
|  | HFD (n=7) | PKO (n=7) | TRF (n=7) | ETRF (n=7) |
| Mean | 11.79 ± 0.906 | 12.71 ± 1.254 | 12.86 ± 0.690 | 12.29 ± 0.488 |

Note: measurement and mean of waist circumference are expressed as centimetre (cm)

Random blood glucose level measured using tail-blood at the end of study according to group

| Sample | Group | | | |
| --- | --- | --- | --- | --- |
|  | HFD (n=7) | PKO (n=7) | TRF (n=7) | ETRF (n=7) |
| Mean | 13.243 ± 1.9637 | 12.743 ± 2.6639 | 16.400 ± 10.2872 | 9.829 ± 2.3027 |

Note: Blood glucose level is expressed as millimole per litre (mmol/L)
